# A shared stress-inflammation signalling architecture underlying chronic disease and multimorbidity

**DOI:** 10.3389/fimmu.2026.1813984

**Published:** 2026-05-07

**Authors:** Wolfgang Kopp

**Affiliations:** Independent Researcher, Graz, Austria

**Keywords:** chronic inflammation, immunometabolism, metabolic stress, multimorbidity, stress signalling, systems immunology

## Abstract

Chronic non-communicable diseases are conventionally classified as distinct clinical entities, yet multimorbidity has become the dominant phenotype in modern populations. Converging experimental, clinical and epidemiological evidence indicates that metabolic overload—characterised by persistent hyperinsulinaemia, insulin resistance and nutrient-driven anabolic signalling—acts as a central amplifier of conserved stress-response systems and chronic low-grade inflammatory signalling. Under contemporary exposome conditions, sustained metabolic stress chronically engages sympathetic and renin–angiotensin–aldosterone signalling, oxidative stress pathways and innate immune activation, establishing a tightly coupled feed-forward network that stabilises pathological states across organ systems. Here, I synthesise mechanistic data from endocrinology, immunometabolism, vascular biology and ageing research to propose a unified stress-signalling architecture linking systemic stress axes to shared intracellular integration hubs. These include inflammatory transcriptional regulators (NF-κB, AP-1), stress-activated kinases (MAPKs), and nutrient- and oxygen-sensing pathways centred on PI3K–Akt–mTOR and HIF-1α, with additional modulation by Notch signalling. When cytoprotective buffering and stress-resolution mechanisms—particularly NRF2 activity, autophagy and mitochondrial resilience—are insufficient, persistent activation of these hubs enforces inflammatory tone, metabolic inflexibility, impaired proteostasis and maladaptive tissue remodelling, providing a mechanistic explanation for phenotypic convergence across cardiometabolic, inflammatory, degenerative and hyperplastic diseases. Importantly, epidemiological and anthropological observations indicate that multimorbidity is not an inevitable consequence of ageing per se, but instead reflects environmentally contingent persistence of stress-response programmes rather than intrinsic ageing trajectories. Framing chronic disease through this integrated metabolic–stress signalling architecture positions multimorbidity as a mismatch phenotype rather than a programmed endpoint of human longevity, and highlights upstream stress regulation and restoration of metabolic resilience as central targets for system-level prevention and intervention.

## 1 Introduction

Non-communicable diseases (NCDs) are traditionally classified as distinct clinical entities. This article presents a hypothesis-generating conceptual synthesis that integrates convergent mechanistic evidence across endocrinology, immunometabolism, vascular biology and ageing research to propose a unified stress-signalling architecture and to derive testable predictions regarding chronic disease emergence and multimorbidity. It is not a systematic review and does not aim to provide exhaustive coverage of the literature. Conventional organ-based classification obscures shared upstream regulatory architectures and reinforces conceptual silos in chronic disease research. However, a growing body of evidence indicates substantial overlap in the endocrine, metabolic and inflammatory disturbances that characterise diverse NCDs (1–4). Across diverse conditions, recurring features such as low-grade inflammation, oxidative and metabolic stress, sympathetic activation and impaired tissue resilience are consistently observed. Despite broad recognition of these shared biological patterns, the mechanistic relationships among systemic stress axes — and their role as coordinated drivers of multiple NCDs — have remained incompletely integrated. This synthesis is motivated by the observation that many chronic diseases share a conserved pattern of neuroendocrine, metabolic, oxidative and innate-immune dysregulation, suggesting a common upstream architecture rather than independent organ-specific etiologies.

Modern environmental exposures, including caloric abundance, circadian disruption, psychosocial load and persistent immune or pollutant stimulation, chronically engage stress pathways that evolved to manage acute threats (5). Throughout this article, ‘metabolic overload’ denotes sustained nutrient and anabolic signalling inputs that chronically exceed cellular energetic demand and restorative capacity; ‘stress signalling’ denotes the integrated activation of neuroendocrine, metabolic, oxidative and innate-immune pathways in response to physiological threat; and ‘network stabilisation’ denotes the acquisition of self-sustaining positive-feedback dynamics that maintain a signalling state beyond the duration of the initiating stimulus. ‘Stress-response pathways’ denotes the conserved neuroendocrine, metabolic, oxidative and innate-immune systems that coordinate physiological adaptation to threat, injury and energetic challenge. ‘Network architecture’ denotes the pattern of regulatory interactions — including bidirectional crosstalk, convergent signalling and feed-forward amplification — among these pathways and their intracellular effectors. ‘Multimorbidity’ denotes the concurrent presence of two or more chronic non-communicable conditions in the same individual, reflecting shared upstream biological drivers rather than coincidental co-occurrence.

Under these conditions, sympathetic and renin–angiotensin–aldosterone system (RAAS) tone increase, innate immune signalling is amplified, and hyperinsulinaemia and oxidative stress emerge as sustained features of physiology (6–10). What has been lacking is a unifying mechanistic framework explaining how activation of individual stress axes propagates across the system and stabilises chronic disease states.

**Figure 1:**
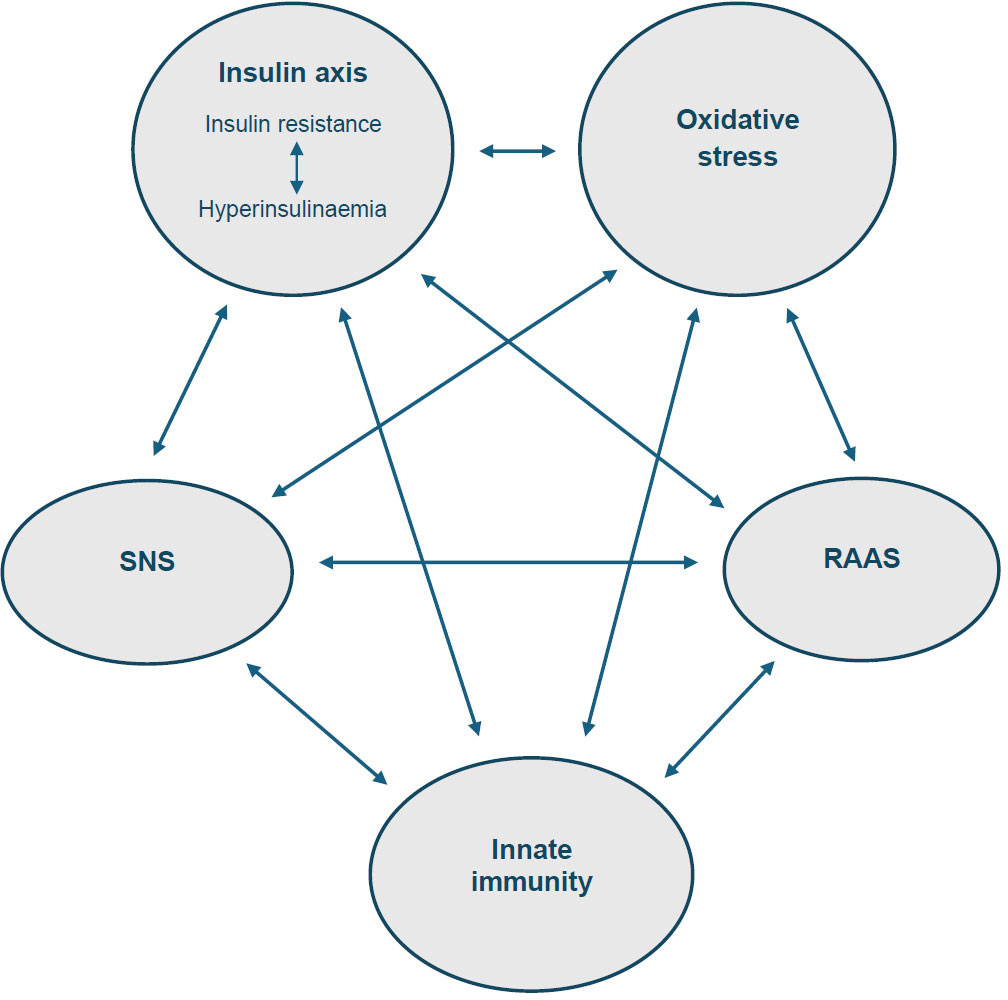
Integrated systemic stress axes in chronic disease. Schematic representation of the major systemic stress axes chronically activated under modern exposome conditions. Persistent sympathetic nervous system (SNS) and renin–angiotensin–aldosterone system (RAAS) activity, metabolic overload with insulin resistance (IR), hyperinsulinaemia (HI), oxidative stress and innate immune activation (INN) form a tightly coupled, self-reinforcing network. Bidirectional interactions among these axes stabilise pathological signalling states and promote multimorbidity across organ systems. The mechanistic basis for these interactions is outlined in Section 2.1.7. Adapted from Kopp (9), licensed under CC BY.

Accumulating evidence indicates that sympathetic activity, RAAS signalling, innate immune activation, hyperinsulinaemia and oxidative stress do not represent parallel or independent abnormalities, but instead constitute elements of a tightly coupled, feed-forward stress network in which activation of one component reinforces the others (**Figure 1**). This integrated endocrine–immune–metabolic architecture has been implicated as a shared upstream driver across a broad spectrum of NCDs, encompassing cardiometabolic, inflammatory, degenerative and hyperplastic conditions (9, 10).

Building on this conceptual foundation, the present synthesis maps how these systemic stress axes converge on conserved intracellular signalling hubs — including nuclear factor κB (NF-κB), activator protein-1 (AP-1), mitogen-activated protein kinases (MAPKs), phosphoinositide 3-kinase–Akt–mechanistic target of rapamycin (PI3K–Akt–mTOR), c-Jun N-terminal kinase (JNK), p38 mitogen-activated protein kinase (p38 MAPK), hypoxia-inducible factor-1α (HIF-1α) and Notch signalling pathway (Notch) — under conditions of insufficient NRF2-mediated cytoprotective buffering (4, 11, 12). Framing chronic disease through this integrated stress-signalling architecture provides a mechanistic basis for multimorbidity and phenotypic convergence across disease categories, and highlights upstream stress regulation as a central target for system-level prevention and intervention.

The synthesis draws on mechanistic studies, experimental models and epidemiological observations across the fields of neuroendocrinology, redox biology, innate immunology, metabolic medicine and ageing research. Evidence was identified through a targeted, hypothesis-driven literature search prioritising studies that elucidate shared regulatory pathways across disease categories. The primary aim is framework construction and hypothesis generation; comprehensive evidence review and formal evidence grading are beyond the scope of this article type.

## 2 Systemic and intracellular stress networks

### 2.1 Systemic stress axes in chronic disease

As outlined above, chronic diseases commonly develop in the setting of persistent activation of conserved physiological stress systems. These systemic axes, which evolved to coordinate acute adaptation to threat, injury and energetic challenge, become maladaptive when chronically engaged under modern environmental conditions. Below, six major stress axes are outlined, together with their functional interactions.

#### 2.1.1 Sympathetic nervous system

The SNS orchestrates rapid defence responses through catecholamine release. Acute activation mobilizes energy, enhances alertness and transiently primes immune responses. Chronic SNS activation, however, promotes endothelial dysfunction, oxidative stress, mitochondrial dysregulation and inflammatory immune polarization (6, 13, 14).

Norepinephrine engages adrenergic receptors across metabolic and immune tissues, activating cAMP/PKA, ERK, JNK and NF-κB signalling (6, 14, 15). Sustained adrenergic tone increases lipolysis, elevates circulating free fatty acids and impairs insulin receptor signalling, thereby contributing to insulin resistance (IR) (16). In parallel, SNS signalling primes innate immune cells and augments cytokine release, linking neurogenic stress to sterile inflammation (17, 18).

#### 2.1.2 Renin–angiotensin–aldosterone system

The RAAS maintains vascular tone, fluid balance and perfusion. Under chronic activation — through obesity, hypertension, hypoxia, inflammation or stress — angiotensin II signalling becomes pathogenic (7, 19, 20).

Angiotensin II activates AT_1_ receptors to stimulate nicotinamide adenine dinucleotide phosphate oxidases, MAPKs, NF-κB and PI3K–Akt pathways, promoting oxidative stress, vascular inflammation and fibrosis (7, 21, 22). In immune and vascular cells, RAAS activation amplifies redox imbalance, inflammasome priming and pro-inflammatory polarization (23, 24). Reciprocal reinforcement between RAAS and sympathetic outflow establishes a feed-forward neurohormonal loop (25).

#### 2.1.3 Oxidative stress and reactive oxygen species

ROS function as essential signalling mediators in physiological adaptation. When ROS generation exceeds antioxidant buffering capacity — owing to mitochondrial dysfunction, RAAS and SNS hyperactivity, hyperglycaemia or inflammatory signalling — chronic oxidative stress ensues (3, 26).

Excess ROS activate redox-sensitive transcriptional programmes, including NF-κB, AP-1, p38, JNK and HIF-1α, while impairing insulin signalling and endothelial nitric oxide (NO) bioavailability (27). Sustained oxidative stress contributes to mitochondrial DNA damage, defective autophagy and cellular senescence, thereby reinforcing inflammatory and fibrotic responses across tissues (27, 28).

#### 2.1.4 Insulin resistance

IR represents a core metabolic stress state arising from nutrient excess, inflammation, oxidative stress, RAAS and SNS activation and ageing (8, 29). IR is mediated by serine phosphorylation of insulin receptor substrate proteins via JNK, IKKβ and mTORC1, mitochondrial dysfunction and lipid intermediate accumulation (30, 31).

Beyond impaired glucose uptake, IR increases hepatic gluconeogenesis, disrupts endothelial function and elevates sympathetic tone, establishing bidirectional interactions with neurohormonal and inflammatory systems (32).

#### 2.1.5 Hyperinsulinaemia

Hyperinsulinaemia arises as a compensatory response to IR but can also emerge independently under chronic dietary stimulation. Sustained insulin hypersecretion — triggered by prolonged exposure to high-glycaemic or insulinotropic diets — may itself precipitate IR, β-cell stress and adipose expansion (33, 34).

Chronic insulin signalling activates PI3K–Akt–mTOR and MAPK pathways, promoting anabolic growth, fibroblast activation and altered immune cell programming (35, 36). Hyperinsulinaemia enhances sympathetic activity, upregulates RAAS, increases oxidative stress and drives adipose inflammation (37). Prolonged insulin excess contributes to proliferative responses, impaired autophagy and accelerated tissue dysfunction (38–40).

#### 2.1.6 Innate immune activation

Innate immune pathways are essential for pathogen defence and tissue repair. Under chronic metabolic, oxidative and neurohormonal stress, however, these circuits become persistently engaged. Toll-like receptor signalling (especially TLR4), inflammasome priming and NF-κB/MAPK cascades are chronically activated by damage-associated molecular patterns, altered lipids, ROS and sympathetic input (41–43).

This sustained activation drives cytokine amplification, macrophage polarization toward pro-inflammatory phenotypes and metabolic reprogramming toward glycolysis, promoting endothelial dysfunction, impaired repair responses and cellular senescence (36, 44). Chronic innate immune engagement thereby reinforces IR, oxidative stress and tissue injury, establishing a self-perpetuating inflammatory state across organ systems.

#### 2.1.7 Integration: a self-reinforcing stress network

These stress axes do not operate independently but form a tightly coupled regulatory network. Sympathetic activation stimulates RAAS and inflammatory pathways; RAAS activity increases oxidative stress and adrenergic tone; ROS amplify inflammatory signalling and IR; and metabolic stress sustains ROS production and innate immune activation (9, 10).

Mechanistic studies demonstrate extensive bidirectional crosstalk among these axes, converging on a shared intracellular signalling core (36, 43–47). Together, these interactions establish a positive-feedback stress loop that maintains chronic activation and drives disease progression (**Figure 1**). At the cellular level, these interacting systemic inputs are integrated by a limited set of conserved intracellular signalling pathways, which translate persistent stress exposure into stable molecular and phenotypic states.

### 2.2 Shared intracellular stress-response pathways

The evidence base supporting the pathway interactions described in this section is heterogeneous. Some relationships are supported by interventional or genetic studies establishing causal directionality — including JNK-mediated serine phosphorylation of IRS proteins as a mechanism of insulin resistance (56, 57), mTORC1-dependent suppression of autophagy (35, 61), and NF-κB-driven cytokine amplification (48, 108). Others are established primarily through mechanistic plausibility in experimental models or epidemiological association, without definitive causal evidence in humans. This distinction is acknowledged throughout and is addressed explicitly in Section 5.6.

Chronic activation of systemic stress axes engages conserved intracellular programmes that normally coordinate acute defence, metabolic flexibility and tissue repair. When activation becomes sustained, however, these signalling nodes enforce a persistent inflammatory tone, drive metabolic reprogramming, impair proteostasis, promote cellular senescence and ultimately contribute to tissue degeneration.

#### 2.2.1 NF-κB and AP-1

NF-κB and AP-1 function as central integrators of inflammatory, metabolic and danger-associated signals. Activated by cytokines, damage-associated molecular patterns (DAMPs), ROS and neurohormonal inputs, these transcription factors regulate gene programmes controlling cytokine production, chemokine expression, adhesion molecules and cell-survival pathways (36, 42, 48).

When chronically activated, these pathways sustain low-grade inflammation, amplify ROS generation, reprogramme cellular metabolism toward glycolysis and suppress mitochondrial biogenesis (49, 50). In metabolic tissues, sustained NF-κB–JNK signalling disrupts insulin receptor substrate function and reinforces IR (51, 52). In structural tissues, prolonged activation promotes fibro-inflammatory remodelling, extracellular-matrix degradation and cellular senescence.

#### 2.2.2 MAPK signalling

The ERK, JNK and p38 MAPK pathways integrate mechanical, oxidative, metabolic and inflammatory stress signals, acting as core transducers of diverse danger cues (27, 53). Acute MAPK activation supports adaptive cell survival, proliferation and tissue repair. When signalling becomes sustained, however, ERK, JNK and p38 drive persistent inflammatory transcription, apoptosis or maladaptive survival programmes, fibroblast activation and extracellular-matrix remodelling (54, 55).

ERK signalling contributes primarily to proliferative remodelling and smooth-muscle hypertrophy, whereas JNK and p38 respond strongly to ROS, cytokines, lipids and DAMPs, coupling metabolic overload to inflammatory gene expression (42). JNK-mediated serine phosphorylation of insulin receptor substrate proteins impairs insulin signalling and reinforces systemic IR (56, 57). Reciprocal cross-activation between MAPKs and NF-κB/AP-1 stabilises chronic inflammatory states and promotes maladaptive remodelling across metabolic, vascular and neural tissues (58).

#### 2.2.3 PI3K–Akt–mTOR pathway

The PI3K–Akt–mTOR axis is a central coordinator of nutrient sensing, protein synthesis, autophagy and cellular survival programmes (35, 59). Under physiological conditions, this pathway promotes anabolic growth when nutrients and energy are abundant. Chronic stimulation by hyperinsulinaemia, IGF-1 and inflammatory mediators, however, results in sustained mTORC1 activation, suppressing autophagy, impairing proteostasis and altering cellular bioenergetics (60, 61).

Persistent PI3K–Akt signalling inhibits FOXO transcription factors, weakening antioxidant defenses and longevity-associated pathways (4). In immune cells, prolonged mTORC1 activity favours pro-inflammatory phenotypes and glycolytic metabolic reprogramming (44). In metabolic tissues, chronic mTOR activation contributes to IR and loss of metabolic flexibility. In neurons, hyperactive mTOR impairs autophagy–lysosomal clearance and promotes protein aggregation, thereby increasing vulnerability to neurodegenerative processes (62).

#### 2.2.4 TLR4/MyD88 danger-sensing axis

Toll-like receptor 4 (TLR4) detects a broad spectrum of pathogen- and damage-associated molecular patterns, including lipids, oxidized LDL, extracellular-matrix fragments and mitochondrial DNA (63). Through MyD88-dependent signalling, TLR4 activates NF-κB, interferon regulatory factors and MAPKs, initiating innate immune responses (55, 64).

In contemporary metabolic and inflammatory environments, TLR4 is chronically stimulated by nutrient excess and tissue injury signals, sustaining cytokine production, inflammasome priming, glycolytic reprogramming and ROS amplification (65). Persistent TLR4 activation thereby maintains sterile inflammation and contributes to IR in metabolic tissues, endothelial dysfunction and neuroinflammatory responses within the central nervous system (8).

#### 2.2.5 Hypoxia-inducible factor-1α and pseudo-hypoxia

HIF-1α orchestrates cellular adaptation to reduced oxygen availability. Under chronic stress conditions, however, HIF-1α can become aberrantly activated even in normoxia as a consequence of mitochondrial dysfunction, oxidative stress, inflammation and neurohormonal signalling — a state often referred to as pseudo-hypoxia (66, 67).

Sustained HIF-1α activity promotes glycolytic reprogramming, suppresses mitochondrial oxidative metabolism, stabilises pro-inflammatory signalling and drives angiogenesis and fibrosis (50, 68). Within the CNS, chronic HIF-1α activation amplifies microglial inflammatory programmes and contributes to neuronal energy failure and neurodegenerative vulnerability (66).

#### 2.2.6 Notch signalling

The Notch pathway regulates cell-fate decisions, progenitor maintenance and tissue repair through contact-dependent signalling (69, 70). Although tightly controlled under physiological conditions, Notch signalling becomes chronically activated in inflammatory, oxidative and metabolically overloaded environments (71). Sustained Notch activity promotes fibroblast activation, smooth-muscle proliferation and epithelial–mesenchymal transition, driving vascular remodelling, adipose inflammation, joint degeneration and stromal hyperplasia (72, 73).

#### 2.2.7 NRF2 cytoprotective programme

The NRF2 pathway coordinates antioxidant defence, detoxification, mitochondrial protection and cytoprotective gene expression. Under acute stress conditions, NRF2 activation preserves redox balance and cellular resilience. In chronic oxidative and inflammatory states, however, NRF2 signalling becomes insufficient owing to sustained redox burden, impaired autophagy and transcriptional repression (3, 28).

Reduced NRF2 activity increases vulnerability to ROS-mediated damage and metabolic stress, accelerating tissue dysfunction and ageing-related trajectories (74). Although excessive NRF2 activation may support tumour survival in specific contexts, most chronic disease states are characterised by inadequate NRF2-mediated cytoprotection rather than pathological overactivation (12).

#### 2.2.8 Integrative view

**Figure 2:**
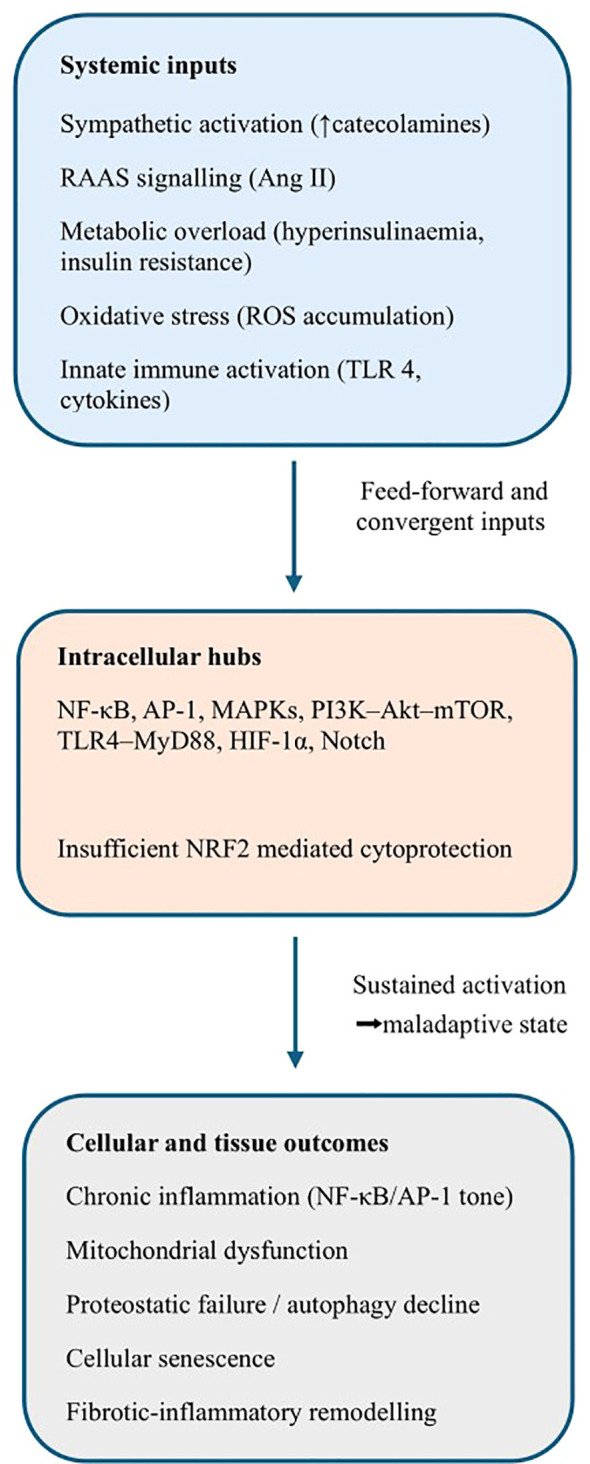
Convergence of systemic stress axes on shared intracellular signalling hubs. Systemic stress inputs converge on a limited set of conserved intracellular signalling pathways, including NF-κB/AP-1, MAPKs, PI3K–Akt–mTOR, TLR4/MyD88, HIF-1α and Notch. Under conditions of insufficient cytoprotective buffering — involving impaired NRF2 activity, reduced autophagy and mitochondrial dysfunction — persistent activation of these hubs enforces inflammatory tone, metabolic inflexibility, proteostatic failure and cellular senescence.

Together, these intracellular programmes constitute a conserved survival architecture that is optimally designed for transient crisis responses. When chronically engaged, however, this network becomes pathogenic, enforcing inflammatory tone, metabolic inflexibility, proteostatic failure, cellular senescence and fibrotic remodelling. This integrated signalling architecture provides a mechanistic link between sustained systemic stress exposure, multimorbidity and age-associated degenerative phenotypes (4, 11) (**Figure 2**).

Among the integrative physiological constraints that govern this intracellular architecture, mitochondrial functional capacity occupies a particularly central position. Mitochondria simultaneously determine the NAD^+^/NADH balance, AMP/ATP ratio and controlled ROS output that regulate the activity of the major stress-termination mechanisms — SIRT1, AMPK, NRF2 and autophagy flux (26, 49, 50, 74). When mitochondrial function is progressively impaired through oxidative mtDNA damage, defective mitophagy or electron-transport-chain uncoupling, resolution capacity is selectively eroded while activation pathways remain accessible to extramitochondrial inputs, providing a plausible mechanistic basis for the transition from adaptive to self-sustaining pathological signalling (4, 26, 49).

**Figure 3:**
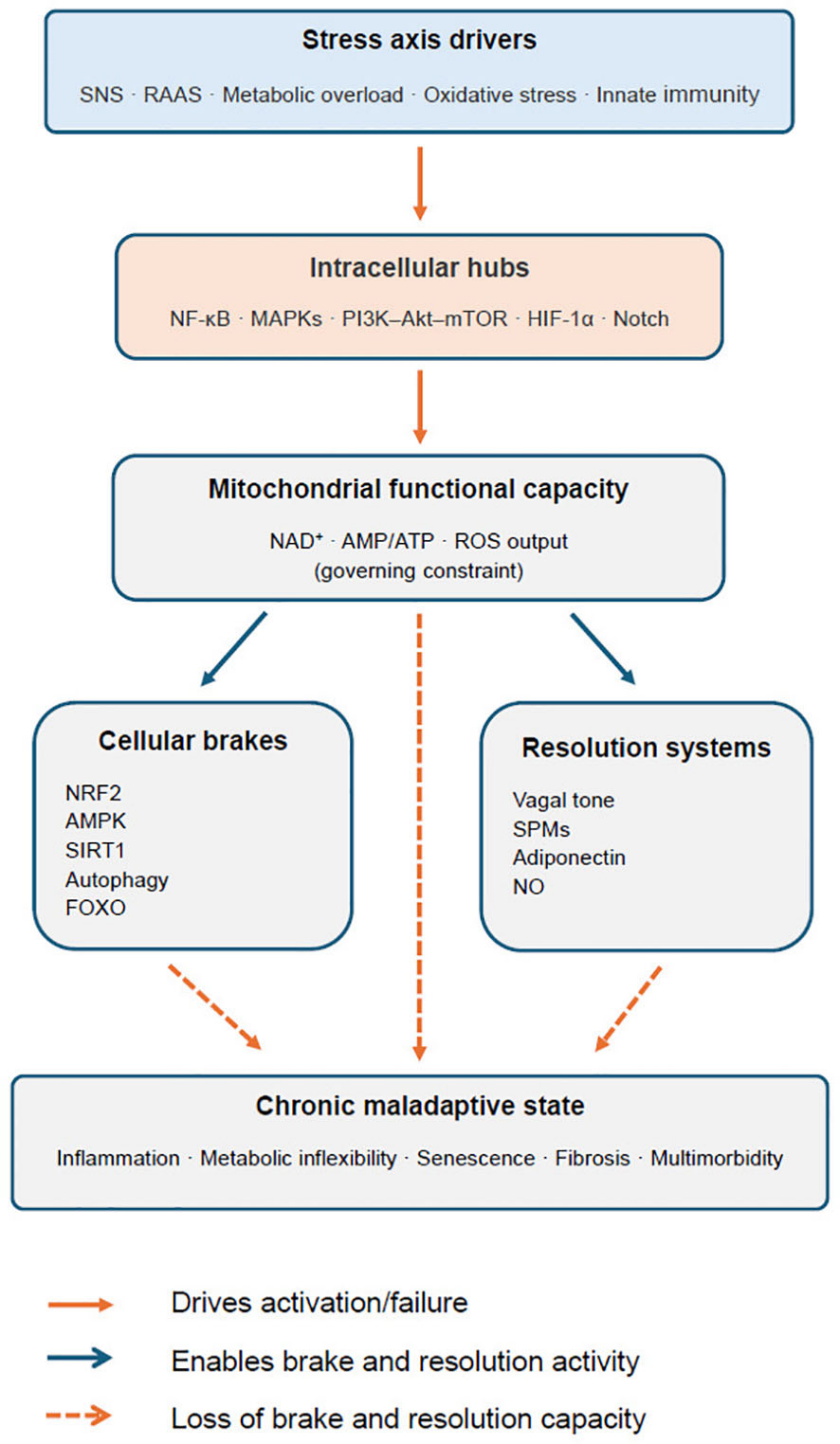
Mitochondrial functional capacity as governing constraint of stress-response resolution. Schematic representation of a stress-signalling architecture in which systemic stress drivers converge on shared intracellular signalling hubs that are functionally constrained by mitochondrial capacity. Mitochondrial function — reflected in NAD^+^ availability, AMP/ATP ratio and controlled ROS output — governs the activity of major cytoprotective and stress-termination systems, including NRF2 signalling, AMPK/SIRT1 pathways and autophagy. Under conditions of sustained stress exposure, progressive impairment of mitochondrial function selectively degrades cellular brakes and resolution systems, while upstream activation pathways remain engaged. The resulting loss of regulatory capacity enables persistent feed-forward signalling, stabilising a chronic maladaptive state characterised by inflammation, metabolic inflexibility, senescence, fibrosis and multimorbidity.

These hubs do not operate as static parallel channels but engage in reciprocal cross-activation that evolves with the duration of stress exposure. Early stress responses may engage NF-κB and MAPK transiently and reversibly; sustained activation progressively recruits mTORC1-dependent suppression of autophagy and HIF-1α-mediated metabolic reprogramming, shifting the system from a recoverable to a self-sustaining state. This temporal progression is governed by the mitochondrial constraint described above and is visualised in **Figure 3**.

#### 2.2.9 Counter-regulatory and resolution systems

A complete account of the proposed architecture requires explicit representation of paired resolution systems. Sympathetic activation is counterbalanced by vagal outflow through the cholinergic anti-inflammatory pathway, which suppresses macrophage NF-κB activity via α7 nicotinic acetylcholine receptors (17, 18); reduced heart-rate variability across cardiometabolic and neurodegenerative disease reflects a resolution deficit within this axis. mTOR-driven anabolic signalling is counterbalanced by AMPK-mediated energy sensing, which promotes autophagy and metabolic flexibility (35, 59). At the immune level, specialised pro-resolving mediators (SPMs) — lipoxins, resolvins, protectins and maresins — actively programme inflammation resolution; their synthesis is impaired in metabolic disease (115), providing a mechanism through which metabolic overload erodes resolution independently of its effects on activation. Chronic disease therefore emerges from the progressive failure of these resolution systems to match the duration of stress-axis engagement.

Clinical evidence for impairment of several resolution-related pathways is substantial. Reduced heart-rate variability — a reliable index of diminished vagal tone — is consistently documented across cardiometabolic, neurodegenerative and chronic inflammatory diseases, and vagal nerve stimulation has demonstrated anti-inflammatory effects in clinical trials in rheumatoid arthritis and inflammatory bowel disease (17, 18). SPM synthesis is impaired in obesity and metabolic syndrome, where elevated omega-6/omega-3 ratios and enzymatic competition reduce lipoxin and resolvin production (115). AMPK responsiveness to energetic signals is blunted in insulin-resistant states, impairing the fasting-induced autophagy and metabolic flexibility that resolution requires (35, 59).

## 3 Disease examples demonstrating framework validity

Chronic diseases that are traditionally classified by organ domain repeatedly engage the same systemic stress axes and intracellular signalling nodes. Although phenotypic manifestations vary with tissue context, the underlying network logic — encompassing neurohormonal activation, metabolic stress, innate immune engagement and impaired cytoprotective capacity — is conserved across disease categories.

### 3.1 Metabolic disease: type 2 diabetes and polycystic ovary syndrome

Type 2 diabetes exemplifies the convergence of metabolic overload, neurohormonal stress and innate immune activation. Chronic hyperinsulinaemia and IR arise from sustained sympathetic and RAAS activity, nutrient excess and adipose tissue inflammation (8, 75). These inputs chronically activate NF-κB, JNK and mTOR signalling, impair insulin-receptor substrate function, reduce mitochondrial efficiency and suppress autophagy (56, 76). Adipose hypoxia and pseudo-hypoxia further amplify inflammatory and metabolic stress circuits, reinforcing systemic IR. Progressive β-cell stress emerges from increased secretory demand, oxidative burden and endoplasmic-reticulum strain, culminating in loss of metabolic flexibility and impaired glucose homeostasis (77).

The framework provides a specific account of the transition from compensated insulin resistance to overt beta-cell failure. Chronically elevated mTORC1 activity progressively erodes beta-cell mitochondrial function through oxidative burden, impaired mitophagy and endoplasmic-reticulum stress (35, 49, 77); once mitochondrial capacity falls below the threshold required to sustain compensatory hypersecretion, secretory decompensation follows — consistent with the observed non-linearity of diabetes onset (33, 56).

Polycystic ovary syndrome reflects the same integrated stress architecture that underlies metabolic disease. Sympathetic overactivity, hyperinsulinaemia, innate immune activation and oxidative stress promote ovarian androgen excess, follicular arrest and systemic metabolic dysfunction (78, 79). In theca cells, sustained sympathetic input and PI3K–Akt–mTOR signalling maintain steroidogenic activation and amplify androgen production (80). These mechanisms closely parallel those observed in type 2 diabetes, underscoring a shared metabolic–neuroendocrine stress network.

Interpretation: Type 2 diabetes and PCOS represent tissue-specific expressions of a common metabolic–neuroendocrine stress network driven by hyperinsulinaemia, IR, inflammatory activation and mitochondrial stress. The mechanistic relationships described are supported primarily by experimental model data and epidemiological association; causal validation in longitudinal human studies remains an important research priority.

### 3.2 Cardiovascular disease

Cardiovascular disease represents a prototypical manifestation of chronic neurohormonal and inflammatory stress. Sustained sympathetic and RAAS activation increases vascular oxidative burden through nicotinamide adenine dinucleotide phosphate oxidases and drives MAPK and NF-κB signalling, leading to endothelial dysfunction, vascular hypertrophy and fibrotic remodelling (7, 21). Hyperinsulinaemia further accelerates vascular remodelling, pro-atherogenic signalling and metabolic inflexibility (31, 40). In the myocardium, HIF-1α-mediated pseudo-hypoxia contributes to maladaptive hypertrophy, mitochondrial impairment and contractile dysfunction (81, 82).

Interpretation: Cardiovascular disease reflects persistent haemodynamic, metabolic and inflammatory stress acting on vascular and myocardial tissues through sustained SNS–RAAS activation, oxidative burden and maladaptive intracellular signalling. Several mechanistic relationships are supported by interventional evidence from clinical trials (ACE inhibitors, beta-blockers, statins); others require further causal validation in humans.

### 3.3 Chronic obstructive pulmonary disease

In COPD, persistent oxidative stress, TLR-driven innate immune activation and chronic NF-κB/AP-1 signalling drive airway inflammation, protease imbalance and accelerated tissue remodelling (83). Environmental oxidants such as cigarette smoke synergise with mitochondrial and inflammatory ROS to sustain DAMP signalling and chronic innate immune activation. Sympathetic and RAAS signalling further amplify oxidative stress and promote fibrotic and vascular remodelling within the lung (84, 85). Impaired NRF2 activity compromises antioxidant and cytoprotective responses, increasing susceptibility to oxidative injury and emphysematous damage (3, 86).

Interpretation: COPD exemplifies chronic innate immune and oxidative stress engagement superimposed on insufficient cytoprotective buffering, resulting in progressive tissue degeneration within the shared stress network. The mechanistic relationships described are supported primarily by experimental model data and epidemiological association; causal validation requires longitudinal human studies.

### 3.4 Autoimmune and inflammatory disease

Autoimmune and inflammatory disorders share a stress-inflammatory architecture characterised by chronic innate immune priming, glycolytic metabolic reprogramming, ROS amplification and neurohumoral activation. Conditions such as rheumatoid arthritis, inflammatory bowel disease and psoriasis exemplify this pattern. Innate immune circuits integrate metabolic overload and neurohormonal inputs, promoting Th17 bias, macrophage activation and cytokine amplification (87, 88). Persistent activation of TLR4–MyD88, NF-κB, MAPK and JAK/STAT pathways sustains cytokine production, inflammasome priming and fibroblast activation (42). Mitochondrial dysfunction reinforces ROS-driven inflammatory tone and stabilises effector phenotypes through Warburg-like metabolic reprogramming (36, 89). Sympathetic and RAAS signalling further erode immune tolerance and intensify both innate and adaptive immune activation (90).

Interpretation: Autoimmune and inflammatory diseases reflect maladaptive persistence of conserved danger-response programmes under chronic metabolic and neurohormonal stress. Several mechanistic relationships are supported by interventional evidence from clinical trials (JAK inhibitors, TNF blockade, vagal nerve stimulation); others warrant prospective human studies with mechanistic endpoints.

### 3.5 Neurodegenerative disorders

Neurodegenerative diseases share convergent biological features including microglial priming, chronic innate immune activation, oxidative stress, mitochondrial decline, impaired autophagy–lysosomal clearance and brain IR. Disorders such as Alzheimer’s disease, Parkinson’s disease, multiple sclerosis and glaucoma exhibit these overlapping mechanisms. Sympathetic and RAAS signalling increase oxidative and inflammatory burden within neural tissues, promoting microglial activation and vascular dysfunction (91, 92). Dysregulated insulin and mTOR signalling impair proteostasis and autophagy, facilitating accumulation of amyloidogenic and other misfolded proteins (93, 94). Mitochondrial dysfunction and ROS-driven metabolic stress further compromise neuronal resilience. In multiple sclerosis, immune dysregulation overlays this metabolic–oxidative stress architecture, accelerating demyelination, glial activation and neurodegenerative progression (95).

The framework also predicts a specific convergence between metabolic disease and neurodegeneration: the same mitochondrial insufficiency and mTOR-dependent autophagy suppression that drives peripheral insulin resistance impairs autophagy-lysosomal clearance of misfolded proteins in neurons (35, 62, 93, 94), providing a mechanistic basis for the epidemiological observation that type 2 diabetes substantially increases Alzheimer’s and Parkinson’s disease risk.

Interpretation: Neurodegenerative diseases arise from failure of metabolic, immune and proteostatic resilience mechanisms under sustained systemic and neuroinflammatory stress. The mechanistic relationships described are supported primarily by experimental model data and epidemiological association; causal validation remains to be confirmed in longitudinal human investigations.

### 3.6 Degenerative disorders: osteoarthritis

Osteoarthritis emerges from the integration of mechanical load, inflammatory signalling, oxidative stress and impaired proteostatic resilience. Chondrocytes exposed to cytokines, ROS and biomechanical strain activate NF-κB, MAPK and Notch pathways, driving catabolic reprogramming, cellular senescence and extracellular-matrix degradation (96, 97). Persistent oxidative burden combined with insufficient NRF2 activity weakens antioxidant defenses, reinforcing inflammatory and metabolic stress (3). Declining autophagy further impairs proteostasis and accelerates structural degeneration within articular cartilage (98).

Interpretation: Osteoarthritis represents a systemic and local stress-response disorder driven by chronic inflammatory, oxidative and mechanical signalling within the shared stress network. The mechanistic relationships described are supported primarily by experimental model data and epidemiological association; causal validation in longitudinal human studies remains an important research priority.

Where degenerative diseases reflect impaired repair capacity under chronic metabolic, inflammatory and mechanical stress, hyperplastic disorders represent the proliferative counterpart of this same network logic, driven by sustained anabolic, inflammatory and neurohormonal cues.

### 3.7 Hyperplastic disorders: benign prostatic hyperplasia

BPH arises from the convergence of metabolic overload, chronic inflammation, oxidative stress and neurohormonal signalling. Hyperinsulinaemia and PI3K–Akt–mTOR activation promote epithelial and stromal proliferation, while innate immune cues and ROS amplify proliferative and fibrotic signalling programmes (99, 100). Sustained sympathetic and RAAS activity enhances stromal remodelling and fibrosis, reinforcing a pro-growth and pro-inflammatory microenvironment within the prostate (101).

Interpretation: BPH reflects a stress-network-driven proliferative remodelling process fueled by hyperinsulinaemia, PI3K–Akt–mTOR activation and inflammatory tone rather than isolated endocrine dysfunction. The mechanistic relationships described are supported primarily by experimental model data and epidemiological association; causal relationships remain to be established in prospective human studies.

### 3.8 Malignant hyperplastic disease (cancer)

Cancer represents a hyperactivated manifestation of the same stress-survival network, augmented by genomic instability and clonal evolution. Persistent activation of inflammatory, metabolic and redox stress programmes — including NF-κB/MAPK signalling, insulin–IGF–PI3K–Akt–mTOR drive and ROS-dependent HIF-1α stabilisation — establishes a proliferative and survival-promoting niche that favours malignant transformation (3, 102, 103). Sympathetic and RAAS signalling further remodel the tumour microenvironment by enhancing angiogenesis, stromal activation and immune suppression, linking systemic stress axes to tumour progression (104, 105). Aberrant NRF2 engagement and mitochondrial rewiring, including UCP2-dependent metabolic flexibility, support resistance to oxidative stress and therapeutic pressure (106, 107).

This stress-integrated view complements — rather than replaces — the somatic mutation theory. While somatic mutations and clonal selection remain essential for malignancy, the coupled stress-response architecture specifies the metabolic, redox and immune context that shapes mutation rates, clonal fitness landscapes and malignant progression.

Interpretation: Cancer represents a context-dependent manifestation of sustained stress-network activation coupled to somatic evolution, rather than a mechanistically isolated disease category. The mechanistic relationships described are supported primarily by experimental model data and epidemiological association; causal validation in longitudinal human studies remains an important research priority.

### 3.9 Interim summary

Across metabolic, vascular, pulmonary, immune, neurodegenerative, degenerative and hyperplastic disorders, a consistent architecture emerges: persistent neuroendocrine and metabolic stress leads to innate immune engagement and oxidative load, converging on conserved intracellular hubs — including NF-κB, MAPKs, PI3K–Akt–mTOR, HIF-1α and Notch — in the setting of insufficient NRF2 activity and impaired autophagy. This shared architecture supports the interpretation of chronic disease as a systems-level stress biology rather than a collection of organ-isolated pathologies.

## 4 Integrative model and evolutionary context

### 4.1 Integrative model: a unified stress-signalling architecture

Converging lines of evidence indicate that many chronic diseases arise from sustained activation of a conserved stress-response network rather than from discrete, organ-restricted failures. Persistent sympathetic and RAAS activity, metabolic overload, hyperinsulinaemia, oxidative stress and innate immune activation form a tightly coupled regulatory ensemble that reinforces itself over time (9, 10, 36, 43).

These systemic drivers impose coordinated demands on a limited set of intracellular control nodes, including NF-κB/AP-1, MAPKs, PI3K–Akt–mTOR, TLR4/MyD88, HIF-1α and Notch, in the context of insufficient NRF2-mediated counter-regulation (86, 108, 109). Together, this signalling architecture governs metabolic allocation, inflammatory tone, repair capacity and cellular fate. When activation becomes chronic, stress programmes that are adaptive in the short term progressively shift toward catabolism, maladaptive growth, impaired proteostasis and loss of tissue resilience (**Figure 3**). Chronic disease thus emerges as a systems-level state of sustained stress signalling rather than as a series of isolated, organ-defined pathologies.

### 4.2 Evolutionary logic: acute survival versus chronic maladaptation

The organisation of human stress physiology reflects deep evolutionary constraints. Stress-survival circuits evolved in environments characterised by severe but intermittent threats, where rapid energy mobilisation, amplified immune defence and prioritisation of immediate survival over long-term maintenance conferred a selective advantage. These responses are best understood within an allostatic framework emphasizing predictive regulation, energetic trade-offs and risk management (5).

From an evolutionary perspective, acute stress responses enabled effective damage control and short-term survival during high-risk episodes, even at the cost of deferred growth, reproduction or long-term health (110). A further dimension of ancestral stress physiology is its temporal structure. Reliable day–night cycles and alternating periods of activity and rest ensured that activation and resolution phases alternated, and that restorative programmes — autophagy, mitochondrial biogenesis and proteostatic repair — were engaged during periods of reduced anabolic drive (4, 110, 111). Temporal organisation is therefore integral to the network’s capacity to complete adaptive cycles.

In modern environments, however, persistent psychosocial load, caloric excess, sedentarism, circadian disruption and chronic low-grade immune stimulation lead to prolonged engagement of these same pathways, rendering them maladaptive (11, 111).

The resulting consequences — mitochondrial dysfunction, loss of proteostasis, cellular senescence and fibro-inflammatory remodelling — are shared hallmarks of ageing and chronic disease (72, 112, 113). Importantly, it is not the nature of the programme that differs, but the duration and contextual persistence of its activation.

### 4.3 Organ-specific outcomes from a shared network

Although a common regulatory logic underlies diverse chronic conditions, tissue vulnerability and phenotypic expression vary according to local metabolic demand, cell-type composition, microenvironmental cues and mechanical constraints. Disease location therefore modifies the output of a shared stress-response programme rather than reflecting independent, disease-specific mechanisms. These context-dependent manifestations of the conserved stress network are summarized in **Table 1**.

**Table 1:** Representative manifestations of the shared stress-signalling architecture across disease categories.

Disease category	Dominant systemic drivers	Key intracellular hubs	Evidence strength	Interpretation within framework
Metabolic (T2D, PCOS)	Hyperinsulinaemia, insulin resistance, SNS activation, inflammation	mTOR, JNK, NF-κB	Strong mechanistic + clinical	Metabolic–neuroendocrine stress network driving insulin resistance and cellular dysfunction
Cardiovascular	RAAS, SNS, oxidative stress	NF-κB, MAPK, PI3K–Akt	Strong mechanistic + clinical	Neurohormonal and redox-driven vascular remodelling
Pulmonary (COPD)	Oxidative stress, innate immune activation	NF-κB, AP-1	Strong experimental + clinical	Persistent innate immune activation and oxidative injury
Autoimmune / inflammatory	Immune activation, metabolic stress	NF-κB, MAPK, JAK/STAT	Strong mechanistic + clinical	Chronic immune activation reinforced by metabolic and neurohormonal inputs
Neurodegenerative	Oxidative stress, mitochondrial dysfunction, insulin resistance	mTOR, HIF-1α, NF-κB	Mechanistic + epidemiological	Impaired proteostasis and neuroinflammation under metabolic stress
Degenerative (osteoarthritis)	Mechanical stress, oxidative stress, inflammation	NF-κB, MAPK, Notch	Experimental + clinical	Chronic stress signalling leading to tissue degeneration
Hyperplastic (BPH)	Hyperinsulinaemia, inflammation	PI3K–Akt–mTOR	Mechanistic + clinical	Proliferative remodelling driven by anabolic and inflammatory signalling
Cancer	Chronic stress signalling + mutations	NF-κB, mTOR, HIF-1α	Strong mechanistic	Stress network provides permissive environment for malignant evolution

Evidence type categories: Interventional = evidence from randomised controlled trials or genetic studies establishing causal directionality. Mechanistic = evidence from experimental models (cell lines, animal studies) with mechanistic plausibility. Epidemiological = association from observational studies. Clinical = evidence from human observational or interventional studies. Evidence levels: Strong = multiple lines of convergent evidence including human interventional data. Moderate = experimental and/or epidemiological evidence without definitive causal human trial data.

### 4.4 Chronic disease as a unified biological category

**Figure 4:**
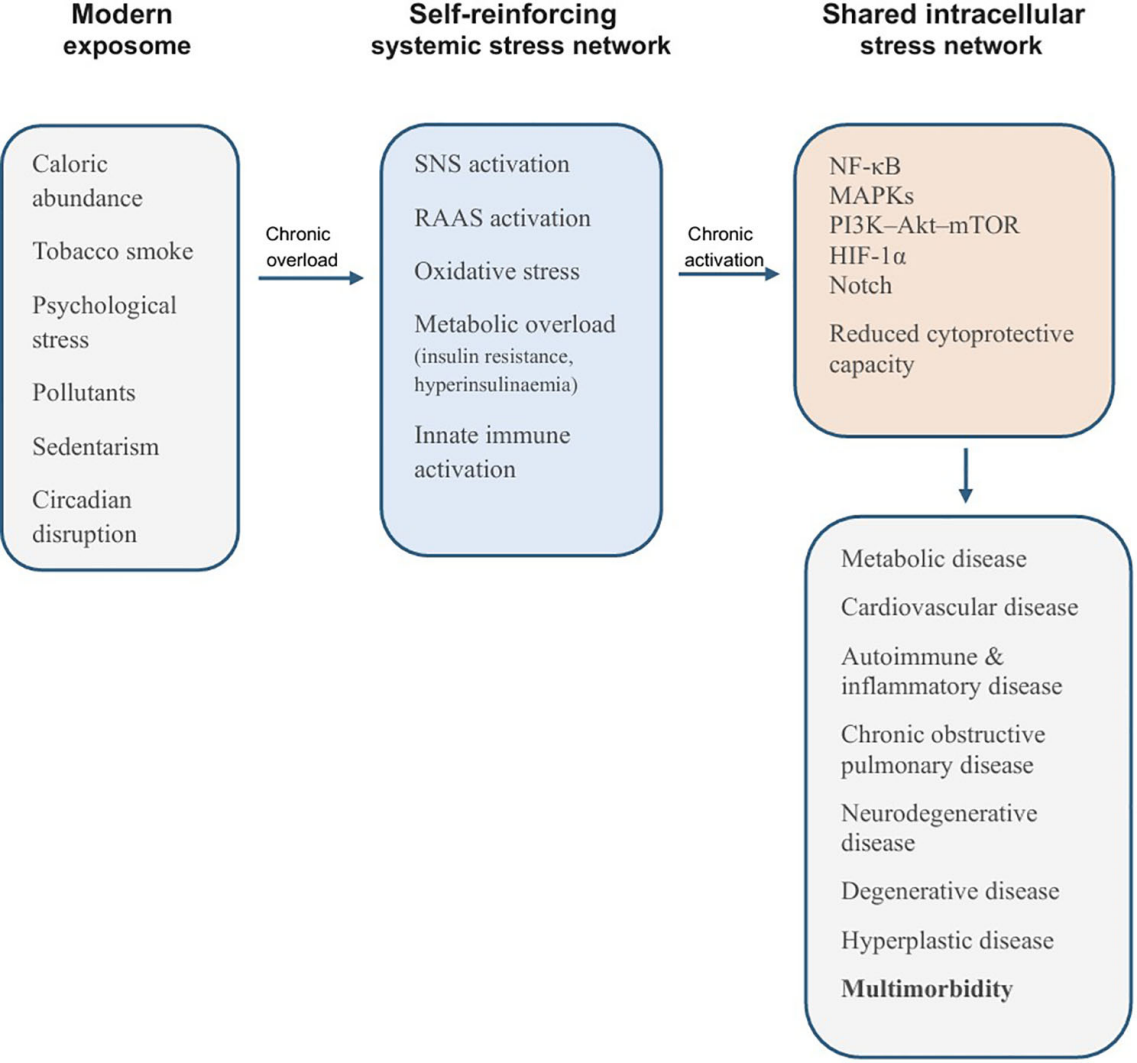
Integrated stress-signalling framework linking modern exposome exposure and multimorbidity. Schematic summary of a systems-level model in which contemporary exposome factors drive sustained activation of systemic stress axes: sympathetic and renin–angiotensin–aldosterone signalling, metabolic overload (insulin resistance and hyperinsulinaemia), oxidative stress and innate immune activation. These inputs converge on shared intracellular integration hubs (NF-κB/AP-1, MAPKs, PI3K–Akt–mTOR, TLR4/MyD88, HIF-1α and Notch). Progressive loss in cytoprotective and stress-termination capacity (reduced NRF2 activity, impaired autophagy and mitochondrial quality control) lowers the threshold for persistent signalling, enabling feed-forward loops that stabilise maladaptive states and promote phenotypic convergence and multimorbidity.

Taken together, this framework positions chronic diseases as context-dependent expressions of a common stress physiology, as summarized in **Figure 4**. Age-associated declines in cytoprotective and stress-termination capacity can lower the threshold for persistent signalling, thereby amplifying chronic disease risk and multimorbidity. Instead of multiple parallel etiologies, a single systems-level model accounts for:

- multimorbidity
- shared molecular and cellular signatures
- overlapping risk factors
- responsiveness to metabolic and stress-axis interventions
- acceleration of pathology under modern exposure patterns

This reconceptualization aligns chronic disease with an allostatic-overload model, in which sustained activation exceeds evolutionary design constraints (5, 43, 114). Ageing and chronic disease thus reflect the same underlying biological logic, expressed at different scales and over different time horizons.

## 5 Discussion

### 5.1 Chronic disease as sustained stress-response activation

Chronic disease is best understood as a durable shift in stress-response set-points. The central question is therefore not which organ fails first, but why stress-response programmes fail to terminate. When neuroendocrine, metabolic and innate-immune inputs remain elevated for prolonged periods, they repeatedly engage the same intracellular signalling pathways (Section 2). Over time, this persistent activation entrenches low-grade inflammation, metabolic inflexibility, impaired proteostasis and mitochondrial decline. Crucially, chronic pathology does not arise from pathway activation per se, but from the progressive failure of regulatory brake systems that normally terminate stress responses. As NRF2-mediated cytoprotection and autophagy become insufficient, damage accumulates and fibrotic remodelling progresses. The governing constraint underlying this transition is mitochondrial functional capacity: because the principal brake systems — NRF2 induction, AMPK activation, SIRT1 activity and autophagy flux — all depend on mitochondrial energetics, progressive mitochondrial impairment selectively degrades resolution capacity while leaving activation pathways intact (26, 49, 50, 74), allowing feed-forward loops to persist beyond the initiating trigger. What emerges is a systems phenotype of maladaptive stress signalling — expressed differently across organs yet governed by a shared biological logic (**Box 1**).

Box 1 **Persistence of stress signalling: brake failure and its bioenergetic basis.**

Governing constraint.

Mitochondrial functional capacity (NAD⁺/NADH balance, AMP/ATP ratio, controlled ROS output) determines whether stress-termination mechanisms can be engaged (26, 49, 50, 74). Progressive mitochondrial impairment selectively erodes resolution capacity while leaving activation pathways accessible to extramitochondrial inputs.

Intracellular brakes (mitochondrially dependent).

NRF2 (12, 74, 86) · AMPK (35, 59) · SIRT1 (4) · FOXO transcription factors (4) · Autophagy flux (39, 61, 62)

System-level resolution pathways.

Vagal/cholinergic anti-inflammatory signalling (α7nAChR) (17, 18) · Specialised pro-resolving mediators (lipoxins, resolvins, protectins, maresins) (115) · Adiponectin · Endothelial NO

Stress-axis drivers.

Sympathetic signalling (6, 13, 14) · RAAS (7, 19, 20) · Hyperinsulinaemia (33, 34, 37) · ROS (3, 27) · DAMPs (63, 64) · Innate immune activation (41, 42, 65) · Circadian disruption (111)

Self-sustaining loops.

NF-κB–cytokine amplification (48, 108) · FFA–ROS–insulin resistance cycling (16, 27, 31) · Hypoxia–HIF-1α stabilisation (66, 78) · mTORC1–autophagy suppression–proteostatic stress (35, 62)

Mechanism.

Under physiological conditions, stress responses terminate once threats resolve, mediated by brake systems dependent on mitochondrial energetics (26, 49, 50, 74). Chronic activation progressively impairs mitochondrial function, depleting the NAD⁺ and ATP availability that brakes require. Simultaneously, impaired SPM synthesis (115) and reduced vagal tone (17, 18) remove systemic resolution signals. When multiple brakes fail in parallel, feed-forward loops become autonomous, converting transient stress exposure into durable tissue remodelling and increased multimorbidity risk (9, 10, 43).

### 5.2 A systems-level explanation for multimorbidity

Conventional models treat diseases as separate processes that simply coexist. Yet the high prevalence of multimorbidity, recurring molecular signatures and shared responses to metabolic and lifestyle interventions challenge this assumption. From a stress-network perspective, co-occurring cardiometabolic, neurodegenerative, inflammatory and degenerative conditions arise from the same upstream drivers acting on conserved intracellular circuitry in the context of impaired signal termination. Multimorbidity therefore reflects a predictable systems outcome of sustained stress signalling and brake failure rather than a coincidence of independent pathologies.

### 5.3 Evolutionary and environmental context

The signalling networks implicated in chronic disease are evolutionarily ancient and tuned for acute survival during intermittent threat, infection or scarcity. Modern environments impose sustained psychosocial load, caloric abundance, circadian disruption, low physical activity and persistent immune stimulation. Under these conditions, circuits adapted for short, intense activation become chronically engaged. This mismatch helps explain the parallel rise of metabolic and inflammatory diseases, accelerated biological ageing and reduced stress resilience.

Importantly, ageing itself does not inevitably lead to chronic disease; rather, maladaptive persistence of stress-survival signalling emerges predominantly under modern, evolutionarily discordant exposome conditions—including caloric excess, tobacco use, chronic psychological stress and circadian disruption—while remaining minimal in evolutionarily concordant (ancestral-like) environments (9, 10). Disruption of circadian organisation — through shift work, artificial light exposure, irregular feeding and fragmented sleep — represents a distinct pathway through which the modern environment impairs stress-response resolution (111). Circadian disruption sustains nocturnal sympathetic tone, suppresses the nocturnal peak of autophagy, reduces AMPK responsiveness during fasting phases and blunts temporal gating of inflammatory cytokine production (49, 50, 111), providing a partial mechanistic explanation for the elevated chronic-disease risk associated with shift work and social jetlag.

Chronic disease is thus not passive wear-and-tear but reflects prolonged activation of adaptive programmes outside their evolutionary design context — a conclusion that applies equally to the circadian, metabolic and psychosocial dimensions of modern life.

### 5.4 Implications for prevention and intervention

If chronic disease reflects sustained activation of systemic stress pathways, targeting upstream axes may offer broader benefits than organ-specific therapies. Relevant strategies include reducing sympathetic and RAAS overactivity, restoring metabolic flexibility and insulin sensitivity, enhancing autophagy and mitochondrial renewal, aligning circadian and sleep rhythms, lowering chronic inflammatory tone and implementing behavioural interventions that reduce allostatic load. Organ-targeted treatments remain essential but may achieve more durable effects when integrated with measures that stabilise systemic stress regulation.

Resolution-targeted interventions directly derivable from the framework include restoration of vagal tone through physical activity (17, 18), omega-3 supplementation to support SPM synthesis (115), AMPK activation through caloric restriction or time-restricted eating (35, 111), and circadian realignment through regularisation of light exposure, sleep and feeding schedules (111). System-level biomarker candidates include heart-rate variability (17), fasting insulin and HOMA-IR (8, 29), circulating 8-isoprostane (3, 27), NAD^+^/NADH ratio in peripheral blood mononuclear cells (26, 74), and GDF15 as a senescence index (112); integrative multi-domain scoring grounded in the framework’s mechanistic structure represents a promising direction for risk stratification (114).

Resolution-targeted interventions with established clinical evidence include: omega-3 fatty acid supplementation, which augments SPM precursor availability and supports SPM-mediated inflammation resolution (115); metformin, whose AMPK-activating and anti-inflammatory effects extend beyond glucose lowering to reduced cardiovascular and cancer risk (35, 111); vagal nerve stimulation, which has shown clinical benefit in rheumatoid arthritis and inflammatory bowel disease (17, 18); and time-restricted eating, which activates AMPK during fasting phases and restores circadian autophagy rhythms (111). These interventions collectively support the view that restoring resolution capacity, rather than solely suppressing activation, represents a mechanistically well-grounded therapeutic strategy.

### 5.5 Conceptual and methodological implications

The proposed framework shares conceptual territory with allostatic load (5), inflammaging (43), immunometabolism (8, 36, 42) and geroscience (4, 11), and is best understood as their integration and extension. Three contributions distinguish it. First, it traces signalling continuity from systemic neuroendocrine inputs through intracellular hubs to tissue outcomes within a single network logic — a cross-level mechanistic integration absent from each existing framework individually. Second, it derives multimorbidity as a predictable systems outcome of a shared upstream architecture (9, 10), rather than acknowledging it descriptively. Third, the mismatch-phenotype framing positions multimorbidity as environmentally contingent rather than age-inevitable (9, 10, 111), with direct implications for prevention.

A unified stress-network framework motivates several research priorities: multi-omics and network-based phenotyping; longitudinal profiling of autonomic, metabolic and inflammatory axes; mechanistic trials targeting upstream pathways; integrated autonomic–metabolic–immune biomarker panels; and dynamic models capturing compensatory and feedback mechanisms. These directions align with emerging work in geroscience and immunometabolism and support classifying chronic diseases as systems-biology entities rather than discrete, organ-defined conditions.

### 5.6 Limitations and future directions

Several limitations of the proposed framework merit explicit acknowledgement. First, the majority of pathway interactions described are established in experimental models — cell lines, rodent studies or short-term human interventions — and their causal relevance to the natural history of chronic disease in humans requires longitudinal validation. Second, the framework does not resolve the directionality of all proposed interactions; some elements designated as drivers may in specific contexts represent compensatory responses to upstream deficits, as discussed in Section 5.1. Third, the integrative architecture necessarily simplifies the substantial tissue- and cell-type-specific heterogeneity of stress signalling — the same pathway may have opposing effects in different cellular contexts. Fourth, the framework does not specify dose–duration thresholds that distinguish adaptive from maladaptive signalling, which is among the most critical unknowns. Addressing these limitations will require longitudinal multi-omics profiling of stress-axis activation and resolution markers, pathway-specific interventional trials with mechanistic endpoints, and formal network analysis to define redundancy, compensatory relationships and reversibility windows. Key evidence gaps and research priorities across disease categories are summarized in **Table 2**.

**Table 2:** Evidence gaps and research priorities within the shared stress-signalling framework.

Disease category	Current evidence base	Key evidence gaps	Research priorities
Metabolic (T2D, PCOS)	Strong mechanistic and interventional evidence for insulin resistance and neuroendocrine drivers	Unresolved causal role of hyperinsulinaemia versus insulin resistance; unclear thresholds for beta-cell decompensation	Longitudinal human studies disentangling insulin resistance and hyperinsulinaemia; intervention trials targeting upstream metabolic stress
Cardiovascular	Strong clinical and interventional evidence for RAAS and SNS pathways	Relative contributions of neuroendocrine and metabolic drivers remain unclear; limited human data on HIF-1α	Integrated intervention studies targeting multiple stress axes; mechanistic human studies on hypoxia signalling
Pulmonary (COPD)	Strong experimental evidence; limited clinical translation	Unclear role of systemic stress axes (SNS/RAAS) in disease progression; insufficient human intervention data	Clinical trials targeting NRF2 and systemic stress modulation; integration of systemic and local disease mechanisms
Autoimmune / inflammatory	Strong interventional evidence (JAK inhibitors, TNF blockade)	Directionality between metabolic stress and immune activation unclear; resolution pathways remain underexplored	Studies targeting resolution biology (SPMs, vagal pathways); integration of metabolic interventions in immune disease
Neurodegenerative	Moderate mechanistic and epidemiological evidence	Peripheral versus central mechanisms unresolved; limited interventional evidence targeting core pathways	Early-phase trials targeting mTOR/autophagy and metabolic dysfunction; longitudinal studies linking metabolic and neurodegenerative trajectories
Degenerative (osteoarthritis)	Moderate evidence from experimental and clinical studies	Interaction between systemic metabolic stress and local mechanical factors unclear	Studies integrating systemic metabolic interventions with local joint pathology; evaluation of Notch signalling in human disease
Hyperplastic (BPH)	Moderate clinical associations with metabolic syndrome	Limited causal evidence linking hyperinsulinaemia to disease progression	Intervention studies targeting metabolic drivers; mechanistic studies of endocrine–proliferative coupling
Cancer	Strong mechanistic evidence for pathway activation; epidemiological associations	Relative contribution of stress signalling versus somatic mutation unclear	Prospective studies targeting systemic stress pathways; integration of metabolic interventions in cancer prevention

### 5.7 Final synthesis

Current evidence supports the view that chronic disease and ageing are context-dependent expressions of a shared adaptive stress programme that becomes maladaptive when persistently engaged. This framework unifies mechanistic and epidemiological findings, accounts for multimorbidity and generates testable predictions for system-level prevention and intervention. The central challenge ahead is translating these insights into scalable diagnostics and targeted strategies that restore stress homeostasis, metabolic flexibility and cellular resilience.

## 6 Conclusion

Chronic diseases are often regarded as distinct pathological entities, yet the synthesis presented here supports a different interpretation: many age-associated disorders represent context-dependent manifestations of a shared stress-response architecture. Persistent activation of sympathetic and renin–angiotensin–aldosterone signalling, oxidative and metabolic stress, hyperinsulinaemia, IR and innate-immune pathways converges on conserved intracellular hubs — including NF-κB, MAPKs, PI3K–Akt–mTOR, TLR4/MyD88, HIF-1α and Notch — in the setting of inadequate NRF2 counter-regulation.

These circuits evolved to manage acute threats but, when chronically activated in modern environments, drive proteostatic decline, mitochondrial dysfunction, senescence and fibro-inflammatory remodelling. This sustained stress-signalling state provides a mechanistic basis for multimorbidity and physiological decline across organ systems. Reframing chronic disease in this way shifts emphasis from isolated organ pathology toward regulation of systemic stress networks, metabolic flexibility and intrinsic defence programmes. Future work should define dose–duration thresholds for maladaptive signalling, delineate network redundancies and develop modulators that selectively constrain chronic activation while preserving acute defence. Translating this systems view into biomarkers, risk stratification tools and targeted interventions will be critical for modifying chronic-disease trajectories and extending health span.
